# Supplementary material for: Chronic sequelae complicate convalescence from both dengue and acute viral respiratory illness
Source: PLoS Negl Trop Dis. 2022 Aug 18;16(8):e0010724. doi: 10.1371/journal.pntd.0010724 (PMC9426910; doi:10.1371/journal.pntd.0010724)
Supplement: S1 Study Questionnaire — (DOCX) [file pntd.0010724.s001.docx]

**Supporting Information**

**S1. MAIDEN content and questions for study participants**

**Study Overview** (this text will be presented to the app user when they first open the app)

1. Wizard Prompt 1
   1. Title: **Welcome!**
   2. “Welcome to MAIDEN! Throughout the next 12 months, we will use this app to collect important research data from you. This information will be used to help improve our understanding of dengue fever.
2. Wizard Prompt 2
   1. Title: **Week 1**
   2. “During Week 1, we will survey you once a day and ask questions about your symptoms. Please stay on the lookout for app notifications and be sure not to miss a day!”
3. Wizard Prompt 3
   1. Title: **Week 2, 3 and 4, 5, 6, 7, 8**
   2. “During Weeks 2-8, we will survey you once a week, and ask you questions about your symptoms and recovery.
4. Wizard Prompt 4
   1. Title: **Months 3, 4, 5, 6, 7, 8, 9, 10, 11, 12**
   2. “After your first 8 weeks, we will begin to survey you monthly.”
5. Wizard Prompt 5
   1. Title: **And you’re done!**

“Thank you for your participation in this study. The information you have provided us will be used to help improve our understanding of dengue fever”.

**Study “Visit” Schedule**

Day 1, 2, 3, 4, 5, 6, 7

Day 14, 21, 28, 35, 42, 49, 56

Then month 3, 4, 5, 6, 7, 8, 9, 10, 11, 12

**Day 1 (**Window of 24 hours for each visit– reminder twice per day)

**Questions on symptoms**

1. “Have you had a fever in the past 24 hours?” Yes or No
   1. If Yes, “What was your highest temperature?”
      1. 37.9 -38.5 °
      2. 38.5 – 40 °C
      3. above 40 °C
      4. I don’t know
2. “Have you experienced any of the following symptoms in the past 24hrs?”
3. Lack of energy: Yes/No
4. Inability to concentrate: Yes/No
5. Poor memory: Yes/No
6. Drowsiness: Yes/No
7. Headache: Yes/No
8. Muscle pain: Yes/No
9. Joint pain: Yes/No
10. Loss of appetite: Yes/No
11. Loss of weight: Yes/No
12. Abdominal pain: Yes/No
13. Diarrhea: Yes/No
14. Nausea: Yes/No
15. Vomiting: Yes/No
16. Rashes: Yes/No
17. Eye Pain: Yes/No
18. Taste Alteration: Yes/No
19. Skin sensitivity: Yes/ No
20. Bleeding: Yes/No

If Yes, please indicate site(s) of bleeding:

- Gums

- Nose

- Blood in urine

- Blood in stool

- Menstrual bleeding (females only)

- Other sites, please specify (free text):

1. Other symptoms, please specify (free text):

SF12v2 Questionnaire

1. In general, would you say your health is:

Excellent/Very Good/Good/Fair/Poor

1. The following questions are about activities you might do during a typical day. Does your health now limit you in these activities? If so how much?
   1. Moderate activities such as moving a table, pushing a vacuum cleaner, bowling or playing golf:

Yes, limited a lot/ Yes, limited a little/No, not limited at all

- 1. Climbing several flights of stairs:

Yes, limited a lot/ Yes, limited a little/No, not limited at all

1. During the past 4 weeks, how much of the time have you had any of the following problems with your work of other regular daily activities as a result of your physical health?
   1. Accomplished less than you would like:

All of the time/Most of the time/Some of the time/A little of the time/None of the time

- 1. Were limited in the kind of work or activities:

All of the time/Most of the time/Some of the time/A little of the time/None of the time

1. During the past week, how much of your time have you had any of the following problems with your work or other regular daily activities as a result of any emotional problems (such as feeling depressed or anxious)?
   1. Accomplished less than you would like:

All of the time/Most of the time/Some of the time/A little of the time/None of the time

- 1. Did work or other activities less carefully than usual:

All of the time/Most of the time/Some of the time/A little of the time/None of the time

1. During the past week, how much did pain interfere with your normal work (including both work outside the home and housework?

Not at all/A little bit/Moderately/Quite a bit/Extremely

1. These questions are about how you feel and how things have been with you during the past week. For each question, please give the one answer that comes closest to the way you have been feeling? How much of the time during the past week
   1. Have you felt calm and peaceful?

All of the time/Most of the time/Some of the time/A little of the time/None of the time

- 1. Do you have a lot of energy?

All of the time/Most of the time/Some of the time/A little of the time/None of the time

- 1. Have you felt downhearted and depressed?

All of the time/Most of the time/Some of the time/A little of the time/None of the time

1. During the past week, how much of the time has your physical health or emotional problems interfered with your social activities (like visiting friends, relatives etc.)

All of the time/Most of the time/Some of the time/A little of the time/None of the time

**Day 2, 3, 4, 5 ,6 (**Window of 24 hours for each visit– reminder twice per day)

1. “Have you had a fever in the past 24 hours?” Yes or No
   1. If Yes, “What was your highest temperature?”
      1. 37.9 -38.5 °
      2. 38.5 – 40 °C
      3. above 40 °C
      4. I don’t know
2. “Have you experienced any of the following symptoms in the past 24hrs?”
   1. Lack of energy: Yes/No
   2. Inability to concentrate: Yes/No
   3. Poor memory: Yes/No
   4. Drowsiness: Yes/No
   5. Headache: Yes/No
   6. Muscle pain: Yes/No
   7. Joint pain: Yes/No
   8. Loss of appetite: Yes/No
   9. Loss of weight: Yes/No
   10. Abdominal pain: Yes/No
   11. Diarrhea: Yes/No
   12. Nausea: Yes/No
   13. Vomiting: Yes/No
   14. Rashes: Yes/No
   15. Eye Pain: Yes/No
   16. Taste Alteration: Yes/No
   17. Skin sensitivity: Yes/ No
   18. Bleeding: Yes/No

If Yes, please indicate site(s) of bleeding:

- Gums

- Nose

- Blood in urine

- Blood in stool

- Menstrual bleeding (females only)

- Other sites, please specify (free text):

1. Other symptoms, please specify (free text):

**Day 7 (**Window of 24 hours for each visit– reminder twice per day)

See Day 1 visit

**Day 14** (Window of 48hrs for each visit – Daily reminder)

1. “Have you had a fever in the past 24 hours?” Yes or No
   1. If Yes, “What was your highest temperature?”
      1. 37.9 -38.5 °
      2. 38.5 – 40 °C
      3. above 40 °C
      4. I don’t know
2. “Are you currently experiencing any of the following symptoms?”
   1. Lack of energy: Yes/No
   2. Inability to concentrate: Yes/No
   3. Poor memory: Yes/No
   4. Drowsiness: Yes/No
   5. Headache: Yes/No
   6. Muscle pain: Yes/No
   7. Joint pain: Yes/No
   8. Loss of appetite: Yes/No
   9. Loss of weight: Yes/No
   10. Abdominal pain: Yes/No
   11. Diarrhea: Yes/No
   12. Nausea: Yes/No
   13. Vomiting: Yes/No
   14. Rashes: Yes/No
   15. Eye pain: Yes/No
   16. Taste Alteration: Yes/No
   17. Skin sensitivity: Yes/ No
   18. Bleeding: Yes/No

If Yes, please indicate site(s) of bleeding:

- Gums

- Nose

- Blood in urine

- Blood in stool

- Menstrual bleeding (females only)

- Other sites, please specify (free text):

- 1. Other symptoms, please specify (free text):

1. Did you miss work or school in the past 1 week due to illness? Yes/No
   1. If “Yes”, “How many days of work or school did you miss?”
      1. 1 day
      2. 2 days
      3. 3 days
      4. 4 days
      5. 5 days
      6. 6 days
      7. Everyday

SF12v2 Questionnaire (See Day 1 visit)

**Day 21, 28, 35, 42, 29** (Window of 48hrs for each visit – Daily reminder)

1. “Are you currently experiencing any of the following symptoms?”
   1. Lack of energy: Yes/No
   2. Inability to concentrate: Yes/No
   3. Poor memory: Yes/No
   4. Drowsiness: Yes/No
   5. Headache: Yes/No
   6. Muscle pain: Yes/No
   7. Joint pain: Yes/No
   8. Loss of appetite: Yes/No
   9. Loss of weight: Yes/No
   10. Abdominal pain: Yes/No
   11. Diarrhea: Yes/No
   12. Nausea: Yes/No
   13. Vomiting: Yes/No
   14. Rashes: Yes/No
   15. Eye pain: Yes/No
   16. Taste Alteration: Yes/No
   17. Skin sensitivity: Yes/ No
   18. Bleeding: Yes/No

If Yes, please indicate site(s) of bleeding:

- Gums

- Nose

- Blood in urine

- Blood in stool

- Menstrual bleeding (females only)

- Other sites, please specify (free text):

- 1. Other symptoms, please specify (free text):

**Day 56** (Window of 48hrs for each visit – Daily reminder)

1. “Are you currently experiencing any of the following symptoms?”
   1. Lack of energy: Yes/No
   2. Inability to concentrate: Yes/No
   3. Poor memory: Yes/No
   4. Drowsiness: Yes/No
   5. Headache: Yes/No
   6. Muscle pain: Yes/No
   7. Joint pain: Yes/No
   8. Loss of appetite: Yes/No
   9. Loss of weight: Yes/No
   10. Abdominal pain: Yes/No
   11. Diarrhea: Yes/No
   12. Nausea: Yes/No
   13. Vomiting: Yes/No
   14. Rashes: Yes/No
   15. Eye pain: Yes/No
   16. Taste Alteration: Yes/No
   17. Skin sensitivity: Yes/ No
   18. Bleeding: Yes/No

If Yes, please indicate site(s) of bleeding:

- Gums

- Nose

- Blood in urine

- Blood in stool

- Menstrual bleeding (females only)

- Other sites, please specify (free text):

- 1. Other symptoms, please specify (free text):

SF12v2 Questionnaire (See Day 1 visit)

**Month 3, 4, 5, 6** (Window of 7 days for each visit – Daily reminder)

1. “Are you currently experiencing any of the following symptoms?”
   1. Lack of energy: Yes/No
   2. Inability to concentrate: Yes/No
   3. Poor memory: Yes/No
   4. Drowsiness: Yes/No
   5. Headache: Yes/No
   6. Muscle pain: Yes/No
   7. Joint pain: Yes/No
   8. Loss of appetite: Yes/No
   9. Loss of weight: Yes/No
   10. Abdominal pain: Yes/No
   11. Diarrhea: Yes/No
   12. Nausea: Yes/No
   13. Vomiting: Yes/No
   14. Rashes: Yes/No
   15. Eye pain: Yes/No
   16. Taste Alteration: Yes/No
   17. Skin sensitivity: Yes/ No
   18. Bleeding: Yes/No

If Yes, please indicate site(s) of bleeding:

- Gums

- Nose

- Blood in urine

- Blood in stool

- Menstrual bleeding (females only)

- Other sites, please specify (free text):

- 1. Other symptoms, please specify (free text):

SF12v2 Questionnaire (See Day 1 visit)

**Month 7** (Window of 7 days for each visit – Daily reminder)

1. “Are you currently experiencing any of the following symptoms?”
   1. Lack of energy: Yes/No
   2. Inability to concentrate: Yes/No
   3. Poor memory: Yes/No
   4. Drowsiness: Yes/No
   5. Headache: Yes/No
   6. Muscle pain: Yes/No
   7. Joint pain: Yes/No
   8. Loss of appetite: Yes/No
   9. Loss of weight: Yes/No
   10. Abdominal pain: Yes/No
   11. Diarrhea: Yes/No
   12. Nausea: Yes/No
   13. Vomiting: Yes/No
   14. Rashes: Yes/No
   15. Eye pain: Yes/No
   16. Taste Alteration: Yes/No
   17. Skin sensitivity: Yes/ No
   18. Bleeding: Yes/No

If Yes, please indicate site(s) of bleeding:

- Gums

- Nose

- Blood in urine

- Blood in stool

- Menstrual bleeding (females only)

- Other sites, please specify (free text):

1. Other symptoms, please specify (free text):

**Month 8** (Window of 7 days for each visit – Daily reminder)

- 1. “Are you currently experiencing any of the following symptoms?”
     1. Lack of energy: Yes/No
     2. Inability to concentrate: Yes/No
     3. Poor memory: Yes/No
     4. Drowsiness: Yes/No
     5. Headache: Yes/No
     6. Muscle pain: Yes/No
     7. Joint pain: Yes/No
     8. Loss of appetite: Yes/No
     9. Loss of weight: Yes/No
     10. Abdominal pain: Yes/No
     11. Diarrhea: Yes/No
     12. Nausea: Yes/No
     13. Vomiting: Yes/No
     14. Rashes: Yes/No
     15. Eye pain: Yes/No
     16. Taste Alteration: Yes/No
     17. Skin sensitivity: Yes/ No
     18. Bleeding: Yes/No

If Yes, please indicate site(s) of bleeding:

- Gums

- Nose

- Blood in urine

- Blood in stool

- Menstrual bleeding (females only)

- Other sites, please specify (free text):

- 1. Other symptoms, please specify (free text):

SF12v2 Questionnaire (See Day 1 visit)

**Month 9** (Window of 7 days for each visit – Daily reminder)

1. “Are you currently experiencing any of the following symptoms?”
   1. Lack of energy: Yes/No
   2. Inability to concentrate: Yes/No
   3. Poor memory: Yes/No
   4. Drowsiness: Yes/No
   5. Headache: Yes/No
   6. Muscle pain: Yes/No
   7. Joint pain: Yes/No
   8. Loss of appetite: Yes/No
   9. Loss of weight: Yes/No
   10. Abdominal pain: Yes/No
   11. Diarrhea: Yes/No
   12. Nausea: Yes/No
   13. Vomiting: Yes/No
   14. Rashes: Yes/No
   15. Eye pain: Yes/No
   16. Taste Alteration: Yes/No
   17. Skin sensitivity: Yes/ No
   18. Bleeding: Yes/No

If Yes, please indicate site(s) of bleeding:

- Gums

- Nose

- Blood in urine

- Blood in stool

- Menstrual bleeding (females only)

- Other sites, please specify (free text):

1. Other symptoms, please specify (free text):

**Month 10** (Window of 7 days for each visit – Daily reminder)

1. “Are you currently experiencing any of the following symptoms?”
2. Lack of energy: Yes/No
3. Inability to concentrate: Yes/No
4. Poor memory: Yes/No
5. Drowsiness: Yes/No
6. Headache: Yes/No
7. Muscle pain: Yes/No
8. Joint pain: Yes/No
9. Loss of appetite: Yes/No
10. Loss of weight: Yes/No
11. Abdominal pain: Yes/No
12. Diarrhea: Yes/No
13. Nausea: Yes/No
14. Vomiting: Yes/No
15. Rashes: Yes/No
16. Eye pain: Yes/No
17. Taste Alteration: Yes/No
18. Skin sensitivity: Yes/ No
19. Bleeding: Yes/No

If Yes, please indicate site(s) of bleeding:

- Gums

- Nose

- Blood in urine

- Blood in stool

- Menstrual bleeding (females only)

- Other sites, please specify (free text):

1. Other symptoms, please specify (free text):

SF12v2 Questionnaire (See Day 1 visit)

**Month 11** (Window of 7 days for each visit – Daily reminder)

1. “Are you currently experiencing any of the following symptoms?”
2. Lack of energy: Yes/No
3. Inability to concentrate: Yes/No
4. Poor memory: Yes/No
5. Drowsiness: Yes/No
6. Headache: Yes/No
7. Muscle pain: Yes/No
8. Joint pain: Yes/No
9. Loss of appetite: Yes/No
10. Loss of weight: Yes/No
11. Abdominal pain: Yes/No
12. Diarrhea: Yes/No
13. Nausea: Yes/No
14. Vomiting: Yes/No
15. Rashes: Yes/No
16. Eye pain: Yes/No
17. Taste Alteration: Yes/No
18. Skin sensitivity: Yes/ No
19. Bleeding: Yes/No

If Yes, please indicate site(s) of bleeding:

- Gums

- Nose

- Blood in urine

- Blood in stool

- Menstrual bleeding (females only)

- Other sites, please specify (free text):

1. Other symptoms, please specify (free text):

**Month 12** (Window of 7 days for each visit – Daily reminder)

1. “Are you currently experiencing any of the following symptoms?”
2. Lack of energy: Yes/No
3. Inability to concentrate: Yes/No
4. Poor memory: Yes/No
5. Drowsiness: Yes/No
6. Headache: Yes/No
7. Muscle pain: Yes/No
8. Joint pain: Yes/No
9. Loss of appetite: Yes/No
10. Loss of weight: Yes/No
11. Abdominal pain: Yes/No
12. Diarrhea: Yes/No
13. Nausea: Yes/No
14. Vomiting: Yes/No
15. Rashes: Yes/No
16. Eye pain: Yes/No
17. Taste Alteration: Yes/No
18. Skin sensitivity: Yes/ No
19. Bleeding: Yes/No

If Yes, please indicate site(s) of bleeding:

- Gums

- Nose

- Blood in urine

- Blood in stool

- Menstrual bleeding (females only)

- Other sites, please specify (free text):

1. Other symptoms, please specify (free text):

SF12v2 Questionnaire (See Day 1 visit)
