# Supplementary material for: Chronic sequelae complicate convalescence from both dengue and acute viral respiratory illness
Source: PLoS Negl Trop Dis. 2022 Aug 18;16(8):e0010724. doi: 10.1371/journal.pntd.0010724 (PMC9426910; doi:10.1371/journal.pntd.0010724)
Supplement: Attachment — Submitted filename: PNTD-D-22-00645_Reviewed.pdf [file pntd.0010724.s002.pdf]

PLOS Neglected Tropical Diseases

Chronic sequelae complicate convalescence from both dengue and acute viral respiratory illness

--Manuscript Draft--

**Manuscript Number:**	PNTD-D-22-00645
**Full Title:**	Chronic sequelae complicate convalescence from both dengue and acute viral respiratory illness
**Short Title:**	Chronic sequelae after dengue and acute viral respiratory illness
**Article Type:**	Research Article
**Keywords:**	Dengue; Viral respiratory infection; chronic sequelae
**Abstract:**	Long Covid has raised awareness of the potentially disabling chronic sequelae that afflicts patients after acute viral infection. Similar syndromes of post-infectious sequelae have also been observed after other viral infections such as dengue, but their true prevalence and functional impact remain poorly defined. We prospectively enrolled 209 patients with acute dengue (n=48; one with severe dengue) and other acute viral respiratory infections (ARI) (n=161), and followed them up for chronic sequelae up to one year post-enrolment, prior to the onset of the Covid-19 pandemic. Baseline demographics and co-morbidities were balanced between both groups except for gender, with more males in the dengue cohort (63% vs 29%, p<0.001). Except for the first visit, data on symptoms were collected remotely using a purpose-built mobile phone application. Mental health outcomes were evaluated using the validated SF-12v2 Health Survey. Almost all patients (95.8% of dengue and 94.4% of ARI patients) experienced at least one symptom of fatigue, somnolence, headache, concentration impairment or memory impairment within the first week of enrolment. Amongst patients with at least 3-months of follow-up, 18.0% in the dengue cohort and 14.6% in the ARI cohort experienced persistent symptoms. The median month-3 SF-12v2 Mental Component Score was lower in patients who remained symptomatic at 3 months and beyond, compared to those whose symptoms fully resolved, indicating that patients who self-reported persistence of symptoms also experienced functionally worse mental health. No statistically significant difference in age, gender distribution or hospitalisation status was observed between those with and without chronic sequelae. Our findings reveal an under-appreciated burden of post-infection chronic sequelae in dengue and ARI patients. They call for studies to define the pathophysiology of this condition, and determine the efficacy of both vaccines as well as antiviral drugs in preventing such sequelae.
**Additional Information:**	
**Question**	**Response**
**Financial Disclosure**<br><br>Enter a financial disclosure statement that describes the sources of funding for the work included in this submission. Review the submission guidelines for detailed requirements. View published research articles from *PLOS NTDs* for specific examples.<br><br>This statement is required for submission and **will appear in the published article** if the submission is accepted. Please make sure it is accurate.	This work was funded by a SingHealth Foundation Research Grant (SHF/FG622P/2016) and the National Research Foundation through the Singapore MIT Alliance for Research and Technology Antimicrobial Resistance Integrative Research Group.

Powered by Editorial Manager® and ProduXion Manager® from Aries Systems Corporation

**Unfunded studies**<br><br>Enter: *The author(s) received no specific funding for this work.*<br><br>**Funded studies**<br><br>Enter a statement with the following details:<br>• Initials of the authors who received each award<br>• Grant numbers awarded to each author<br>• The full name of each funder<br>• URL of each funder website<br>• Did the sponsors or funders play any role in the study design, data collection and analysis, decision to publish, or preparation of the manuscript?<br>• **NO** - Include this sentence at the end of your statement: *The funders had no role in study design, data collection and analysis, decision to publish, or preparation of the manuscript.*<br>• **YES** - Specify the role(s) played.<br><br>* typeset	
**Competing Interests**<br><br>Use the instructions below to enter a competing interest statement for this submission. On behalf of all authors, disclose any competing interests that could be perceived to bias this work—acknowledging all financial support and any other relevant financial or non-financial competing interests.<br><br>This statement **will appear in the published article** if the submission is accepted. Please make sure it is accurate. View published research articles from *PLOS NTDs* for specific examples.	The authors have declared that no competing interests exist.

Powered by Editorial Manager® and ProduXion Manager® from Aries Systems Corporation

**NO authors have competing interests**<br><br>Enter: *The authors have declared that no competing interests exist.*<br><br>**Authors with competing interests**<br><br>Enter competing interest details beginning with this statement:<br><br>*I have read the journal's policy and the authors of this manuscript have the following competing interests: [insert competing interests here]*<br><br>* typeset<br><br>This statement is required for submission and **will appear in the published article** if the submission is accepted. Please make sure it is accurate and that any funding sources listed in your Funding Information later in the submission form are also declared in your Financial Disclosure statement.	
**Data Availability**<br><br>Authors are required to make all data underlying the findings described fully available, without restriction, and from the time of publication. PLOS allows rare exceptions to address legal and ethical concerns. See the PLOS Data Policy and FAQ for detailed information.	No - some restrictions will apply

Powered by Editorial Manager® and ProduXion Manager® from Aries Systems Corporation

A Data Availability Statement describing where the data can be found is required at submission. Your answers to this question constitute the Data Availability Statement and **will be published in the article**, if accepted.<br><br>**Important:** Stating 'data available on request from the author' is not sufficient. If your data are only available upon request, select 'No' for the first question and explain your exceptional situation in the text box.<br><br>Do the authors confirm that all data underlying the findings described in their manuscript are fully available without restriction?	
**Describe where the data may be found in full sentences. If you are copying our sample text, replace any instances of XXX with the appropriate details.**<br><br>• If the data are **held or will be held in a public repository**, include URLs, accession numbers or DOIs. If this information will only be available after acceptance, indicate this by ticking the box below. For example: *All XXX files are available from the XXX database (accession number(s) XXX, XXX.*).<br>• If the data are all contained **within the manuscript and/or Supporting Information files**, enter the following: *All relevant data are within the manuscript and its Supporting Information files.*<br>• If neither of these applies but you are able to provide **details of access elsewhere**, with or without limitations, please do so. For example:	Data cannot be shared publicly because of institutional data regulations on confidential data sharing. Please contact the corresponding author if access to data is required.

Data cannot be shared publicly because of [XXX]. Data are available from the XXX Institutional Data Access / Ethics Committee (contact via XXX) for researchers who meet the criteria for access to confidential data.<br><br>*The data underlying the results presented in the study are available from (include the name of the third party and contact information or URL).*<br>• This text is appropriate if the data are owned by a third party and authors do not have permission to share the data.<br><br>* typeset	
Additional data availability information:	

Powered by Editorial Manager® and ProduXion Manager® from Aries Systems Corporation

Manuscript Click here to access/download;Manuscript;Kalimuddin et al.docx

Chronic sequelae complicate convalescence from both dengue and acute viral respiratory illness

Shirin Kalimuddin[1,2*], Yii Ean Teh[1], Liang En Wee[1], Shay Paintal[3], Ram Sasisekharan[4,5], Jenny G Low[1,2,6], Sujata K Sheth[7], Eng Eong Ooi[2,6,8*]

1. Department of Infectious Diseases, Singapore General Hospital, Singapore
2. Program in Emerging Infectious Diseases, Duke-NUS Medical School, Singapore
3. Accio Systems Pte Ltd, Singapore
4. Antimicrobial Resistance Interdisciplinary Research Group, Singapore-MIT Alliance for Research and Technology, Singapore
5. Department of Biological Engineering, Massachusetts Institute of Technology, USA
6. Viral Research and Experimental Medicine Centre, SingHealth Duke-NUS Academic Medical Centre, Singapore
7. Department of Emergency Medicine, Changi General Hospital, Singapore
8. Saw Swee Hock School of Public Health, National University of Singapore, Singapore

*Correspondence to:

Shirin Kalimuddin, email: shirin.kalimuddin@singhealth.com.sg

Eng Eong Ooi, email: engeong.ooi@duke-nus.edu.sg

Abstract

Long Covid has raised awareness of the potentially disabling chronic sequelae that afflicts patients after acute viral infection. Similar syndromes of post-infectious sequelae have also been observed after other viral infections such as dengue, but their true prevalence and functional impact remain poorly defined. We prospectively enrolled 209 patients with acute dengue (n=48; one with severe dengue) and other acute viral respiratory infections (ARI) (n=161), and followed them up for chronic sequelae up to one year post-enrolment, prior to the onset of the Covid-19 pandemic. Baseline demographics and co-morbidities were balanced between both groups except for gender, with more males in the dengue cohort (63% vs 29%, $p<0.001$). Except for the first visit, data on symptoms were collected remotely using a purpose-built mobile phone application. Mental health outcomes were evaluated using the validated SF-12v2 Health Survey. Almost all patients (95.8% of dengue and 94.4% of ARI patients) experienced at least one symptom of fatigue, somnolence, headache, concentration impairment or memory impairment within the first week of enrolment. Amongst patients with at least 3-months of follow-up, 18.0% in the dengue cohort and 14.6% in the ARI cohort experienced persistent symptoms. The median month-3 SF-12v2 Mental Component Score was lower in patients who remained symptomatic at 3 months and beyond, compared to those whose symptoms fully resolved, indicating that patients who self-reported persistence of symptoms also experienced functionally worse mental health. No statistically significant difference in age, gender distribution or hospitalisation status was observed between those with and without chronic sequelae. Our findings reveal an under-appreciated burden of post-infection chronic sequelae in dengue and ARI patients. They call for studies to define the pathophysiology of this condition, and determine the efficacy of both vaccines as well as antiviral drugs in preventing such sequelae.

Word count: 291

Introduction

As the Coronavirus Disease 2019 (Covid-19) pandemic continues to affect millions around the world, there has been increasing awareness on the potentially disabling syndrome of long Covid. Long Covid afflicts a significant proportion of patients after convalescence from Severe Acute Respiratory Syndrome Coronavirus 2 (SARS-CoV-2) infection, and includes symptoms such as fatigue, “brain fog” and even depression, which persist for 3 months or more acute infection (1-3). Yet, this syndrome of post-infectious chronic sequelae is not confined exclusively to SARS-CoV-2, but has also been observed after convalescence from other acute viral infections, one of which is dengue (4).

Dengue is an acute mosquito-borne viral illness that afflicts an estimated one hundred million people annually (5, 6). There is no licensed anti-dengue therapeutic, and the only currently licensed dengue vaccine has safety and efficacy concerns (7). The acute symptoms of infection usually last 7-10 days, and range from self-limiting undifferentiated fever, to more severe and potentially fatal dengue haemorrhagic fever and shock (5). Much less however, is understood about post-dengue chronic sequelae. Although symptoms such as fatigue and cognitive impairment have been described post-infection, many of these observations were made retrospectively and thus subject to recall bias, or were derived after a relatively short period of study follow-up (8-12). As such, the true prevalence and duration of post-dengue chronic sequelae remain poorly defined. Such sequelae would not only have a direct impact on patient convalescence, they would also detrimentally affect the economic productivity of many individuals living in dengue endemic areas. Consequently, the true societal and economic impact of dengue is likely underestimated (13).

To understand the true prevalence of post-dengue chronic sequelae, we designed a Mobile-phone Application for Information extraction in Dengue (MAIDEN). The use of MAIDEN enabled data collection to be conducted remotely, removing the need for frequent in-person

study visits, thus minimising study costs and avoiding high participant drop-out rates often associated with large prospective studies. In addition, we also took advantage of MAIDEN to explore the impact of chronic sequelae in patients with acute viral respiratory infections (ARI), another highly prevalent group of acute viral infections prior to the emergence of SARS-CoV-2, but in which the occurrence of long-term sequelae is even more poorly defined. We prospectively enrolled two separate cohorts of patients with confirmed dengue and ARI and followed them for up to one-year post-illness onset. Our findings suggest that chronic sequelae such as fatigue and central nervous system (CNS)-related symptoms are prevalent in adults after acute dengue, and also affect a sizeable proportion of patients post-ARI.

Methods

Study Participants

Patients were recruited from the inpatient wards, emergency department and staff healthcare clinic of two hospitals in Singapore, between 20 September 2017 and 29 March 2019.

Patients were divided into two cohorts: acute dengue and those with ARI. Patients were included in the dengue cohort if they had a confirmed diagnosis of acute dengue based on compatible clinical features, positive serum dengue NS1 antigen and/or dengue virus (DENV) polymerase chain reaction (PCR). Patients were included in the ARI cohort if they presented with symptoms compatible with a viral upper respiratory tract infection (fever with rhinorrhea, sore throat or cough) or had a viral respiratory tract infection confirmed by respiratory virus multiplex PCR from a respiratory sample within 48 hours prior to enrolment. All patients enrolled had to have access to a mobile phone with internet capability. In both the dengue and ARI cohorts, patients were excluded if they had active malignancy, neurological, rheumatological or psychiatric conditions, dementia or cognitive impairment, concomitant bacterial infection, pneumonia, surgery within the preceding 3 months, previous hospitalization

in the past 6 months, were receiving immunosuppressive therapy, or had chronic viral infection such as human immunodeficiency virus or hepatitis B or C. Written informed consent was obtained from all patients and the study was approved by the local institutional ethics committee (Ref: CIRB 2017/2308).

Laboratory methods

Serum dengue NS1 was measured using a commercially available single step EIA (Platelia™ Dengue NS1 Antigen, BioRad). DENV genome was detected using a multiplex real-time polymerase chain reaction (PCR) (FTD Zika/Dengue/Chik, Fast Track Diagnostics, Luxembourg) on RNA. Respiratory virus multiplex PCR was performed using an in-house protocol based on the Anyplex™ II RV16 respiratory virus panel (Seegne Inc.), and included the following viruses: respiratory syncytial viruses A and B, influenza A and B, parainfluenza viruses 1-4, metapneumovirus, rhinovirus, human coronavirus OC43, 229E and NL63, adenovirus, human enterovirus and human bocavirus 1-4.

Clinical data collection

At enrolment after informed consent, relevant demographic and clinical data were collected from patients' medical records. Patients were sent a web link via e-mail to download the MAIDEN application onto their mobile phone, as well as a unique user-ID and password to enable them to access the application. A member of the study team then provided a short tutorial on how to use MAIDEN (Fig 1A). At each visit, patients would enter information regarding symptoms experienced (including the absence or presence of fatigue, somnolence, headache, concentration impairment and/or memory impairment) and self-reported overall health into MAIDEN (see Supporting Information S1 for the full list of questions). The overall health assessments were adapted from the SF-12v2® health survey, which is a validated tool

for self-reported physical and mental wellbeing measurement (14-17). Briefly, the SF-12v2® consists of 12 questions that measure eight health domains to assess physical and mental health. Specifically, mental health-related scales include Vitality (VT), Social Functioning (SF), Role Emotional (RE), and Mental Health (MH). Mental Composite Scores (MCS) were calculated using the weighted means of each mental health domain, with a score of 50 considered the population average. Thus, a score below 50 would indicate worse mental health than the expected average, and a score above 50 would indicate better mental health.

Patients were followed-up daily for the first 7 days, weekly from weeks 2 - 8, and then monthly from month-3 onwards, up to one-year following acute illness. Except for the first visit at study enrolment, patients were not required to attend the study site in-person, and would be sent reminders by either short messaging system (SMS) or push notification to enter data remotely into MAIDEN when each study "visit" was due. If patients encountered any technical difficulties or had questions about the study, they could contact the study team directly either via phone call or email, or through a direct messaging system within MAIDEN. All data entered into MAIDEN was stored on a secure cloud server and downloaded onto a data management platform for analysis.

Definitions

We defined "chronic sequelae" as symptoms (fatigue, somnolence, headache, concentration impairment or memory impairment) lasting for at least 3-months post-infection. A patient's symptoms were considered resolved if they did not report any symptoms for at least 2-months after the last date of symptoms reported, and were considered lost to follow-up if they did not enter any data into MAIDEN for more than a 2-month period.

Statistical Analysis

Differences in demographic features, co-morbidities, prevalence of symptoms at enrolment and MCS between the dengue and ARI cohorts, and those with and without chronic sequelae were analysed using the Fisher's exact test for categorical variables or Mann-Whitney test for continuous variables. Time to resolution of symptoms in both the dengue and ARI cohorts were analysed using Kaplan-Meier analyses. The SF12v2 MCS was calculated using the Optum® PRO CoRE software. P-values <0.05 were considered statistically significant. All statistical analyses were performed using GraphPad Prism version 9.2.0.

Results

A total of 209 patients were enrolled during the study period, with 48 patients in the dengue cohort, and 161 patients in the ARI cohort. 81.3% (39/48) of dengue patients and 98.1% (158/161) of ARI patients completed at least 3 months of study follow-up (Fig 1B). Table 1 shows the demographic and clinical profile of both patient cohorts. Baseline demographics such as age, ethnicity and co-morbidities were balanced between both groups, except for gender, with more males in the dengue cohort (63% vs. 29%, p<0.001). More patients in the dengue cohort were hospitalized compared to those in the ARI cohort (77.1 % vs 18.1%, p<0.001), reflecting differences in clinical management between these two disease types. Only one patient had severe dengue, as defined by the World Health Organization (WHO) 2009 dengue classification scheme (18). Within the ARI cohort, the specific viral etiology was tested for in 29 patients. Among these patients, influenza virus and rhinovirus were the most common etiologies identified (Table 1).

	Dengue Cohort (n=48)	ARI Cohort (n=161)	p-value
**Demographics**			
Age			
Median (range), years	37.0 (21 - 68)	34.0 (22 - 79)	0.39
Male sex, no. (%)	30 (62.5)	46 (28.6)	**<0.001**
Ethnicity, no. (%)			0.21
Chinese	29 (60.4)	77 (47.8)	
Malay	12 (25.0)	36 (22.4)	
Indian	3 (6.3)	21 (13.0)	
Others	4 (33.3)	27 (16.8)	
Co-morbidities, no. (%)			
Diabetes mellitus	2 (4.2)	5 (3.1)	0.72
Hypertension	4 (8.3)	16 (9.9)	0.74
Ischemic heart disease	2 (4.2)	2 (1.2)	0.19
**Disposition**			
Hospitalised, no (%)	37 (77.1)	29 (18.0)	**<0.001**
**Study Compliance**			
Completed at least 3 months of study follow-up, no (%)	39 (81.3)	158 (98.1)	**<0.001**
**Viral Etiology**			
Severe dengue, no. (%)	1 (2.0)	-	NA
Respiratory virus type, no. (%)*			
Influenza A & B	-	14 (48.3)	
Rhinovirus	-	6 (20.7)	
Adenovirus	-	4 (13.8)	
Human coronavirus (OC43)	-	4 (13.8)	
Parainfluenza	-	3 (10.3)	
Human coronavirus (NL63)	-	1 (3.4)	
Enterovirus	-	1 (3.4)	
**Clinical Features**			
Symptom type at presentation, no (%)			
Any symptom**	46 (95.8)	152 (94.4)	1.00
Fatigue	38 (79.2)	136 (84.5)	0.51
Somnolence	32 (66.7)	93 (57.8)	0.32
Headache	29 (60.4)	119 (74.0)	0.07
Concentration impairment	29 (60.4)	95 (59.0)	0.86
Memory impairment	13 (27.1)	28 (17.4)	0.15

Table 1. Demographics and clinical features of the study cohort. *Within the ARI cohort, the specific viral etiology was known in 29 patients. The percentage reported is out of a total of 29 patients. Of these 29 patients, 4 patients tested positive for two different viruses concurrently on multiplex PCR – influenza and adenovirus (n=1), influenza and human coronavirus OC43 (n=1), parainfluenza and respiratory syncytial virus (n=1), and parainfluenza and enterovirus virus (n=1). **Any one of fatigue, somnolence, headache, concentration impairment or memory impairment.

Almost all patients (95.8% of dengue and 94.4% of ARI patients) experienced fatigue or at least one other CNS-related symptom (somnolence, headache, concentration impairment

and/or memory impairment) within the first week after enrolment. Fatigue and somnolence were the most commonly reported symptoms in the dengue cohort, while lethargy and headache were most common in the ARI cohort (Table 1). Amongst patients with at least 3-months of follow-up, 18.0% in the dengue cohort and 14.6% in the ARI cohort experienced chronic sequelae (Table 2). In the dengue cohort, fatigue, concentration impairment and memory impairment were the most commonly reported chronic symptoms, while fatigue and headache were most prevalent in the ARI cohort. Kaplan-Meier analysis indicated that fatigue and CNS-related symptoms resolved within weeks of convalescence in the majority of dengue patients. However, the rate of full recovery then plateaued and even persisted in some patients until the end of our observation period (Fig 2A). Similar trends were observed in patients with ARI (Fig 2B). No statistically significant difference in age, gender distribution or hospitalisation status was observed between those with and without chronic sequelae (Table 3).

	Dengue Group (n =39)	ARI Cohort (n=158)	p-value
**Chronic Sequelae**			
Any symptom persisting $\geq$ 3 months*, no. (%)	7 (18.0)	23 (14.5)	0.62
Symptom type at month 3, no (%)			
Fatigue	4 (10.3)	15 (9.5)	1.00
Somnolence	1 (2.6)	10 (6.3)	0.47
Headache	2 (5.1)	13 (8.2)	0.74
Concentration impairment	4 (10.3)	10 (6.3)	0.48
Memory impairment	4 (10.3)	8 (5.1)	0.25

Table 2. Symptoms in patients with chronic sequelae. Analysis performed on patients with at least 3 months of study follow-up (dengue: n=39, ARI: n=158). **Any one of fatigue, somnolence, headache, concentration impairment or memory impairment.

	Dengue Group (n=39)			ARI Group (n=158)			All (n=197)		
	Chronic sequelae			Chronic sequelae			Chronic sequelae		
	Absent (n=32)	Present (n=7)	p-value	Absent (n=135)	Present (n=23)	p-value	Absent (n=167)	Present (n=30)	p-value
**Age, years**									
Median (range)	38.5 (21-68)	51 (24-60)	0.50	35.0 (23-67)	31.0 (22-64)	0.27	35.0 (21-68)	31.5 (22-64)	0.72
**Gender**									
Male sex, no. (%)	21 (65.6)	3 (42.9)	0.40	40 (29.6)	6 (26.1)	0.81	61 (36.5)	9 (30.0)	0.54
**Disposition**									
Hospitalised, no. (%)	26 (81.3)	6 (85.7)	1.00	20 (14.8)	7 (30.4)	0.08	46 (27.5)	13 (43.3)	0.09

Table 3. Demographics of patients with and without chronic sequelae. Chronic sequelae is defined as persistence of symptoms (any one of fatigue, somnolence, headache, concentration impairment or memory impairment) for at least 3 months.

In both cohorts, the median month-3 MCS was lower in patients who remained symptomatic at 3 months and beyond, compared to those whose symptoms fully resolved (Fig 3), indicating that patients who self-reported persistence of symptoms beyond 3 months also experienced functionally worse mental health.

Overall, our findings indicate that a significant proportion of both dengue and ARI patients experienced chronic sequelae, and the persistence of such chronic sequelae translated to overall worse mental health outcomes.

Discussion

To date, few studies have examined the long-terms health effects of either acute dengue or non-SARS-CoV-2 ARI, making it difficult to quantify the true societal and economic impact of such acute viral infections. In this prospective study of over 200 patients, we found that a significant proportion of dengue, and even more unexpectedly ARI patients, continue to experience fatigue and CNS-related chronic sequelae for several months after resolution of the acute infection, not dissimilar to long Covid sufferers (1, 2).

At present, it still remains unclear what the drivers of such chronic sequelae are, and unlike Covid-19, little research has focused on the mechanistic causes of chronic sequelae post-

dengue. This is even less so for acute respiratory viral infections such as influenza and adenovirus. In one study of dengue patients, an association was found between the FcγRIIa (FcγRIIa-131HH) gene polymorphism, the presence of autoimmune markers and symptom persistence (9). Apart from this report, no other studies to date explored the causes of post-dengue chronic sequelae.

It is possible that aberrant activation of the innate immune response to the original viral stimuli may result in chronic inflammation and subsequent long term tissue damage and immune exhaustion, which may then in turn drive the development of chronic sequelae. Indeed, we have recently shown that increased baseline expression of genes associated with T-cell exhaustion was associated with the development of fatigue after SARS-CoV-2 mRNA vaccination (manuscript in press); such immune dysregulation has also been associated with the development of chronic fatigue syndrome/myalgic encephalomyelitis (19-21). In the context of long Covid, multiple mechanisms have been postulated, including chronic inflammation, antigen persistence, latent virus reactivation and development of auto-antibodies (2-4, 22). It is likely that some, if not all of these mechanisms may also apply to the development of chronic sequelae after other acute viral infections. The prevalence of such chronic sequelae and their functional impact on mental well-being underscore the need for more in-depth research to understand the specific drivers of such post-infection chronic outcomes.

We used MAIDEN as a study tool for remote data-collection in order to overcome the high financial costs and participant drop-out rates often associated with large prospective studies. We reasoned that remote data collection would promote better compliance from study participants in view of the convenience afforded through remote data collection. Indeed, overall study compliance was good with over 90% of patients completing at least 3 months of study follow-up. Besides improving study visit compliance, mobile data collection also allows for

immediate pooling of data into a centralized data warehouse, making real-time data analysis possible. Given these benefits, and the fact that over 80% of the world's population own a smartphone (23), we envisage that mobile phone applications such as MAIDEN could be a useful adjunct tool not only in the conduct of observational cohort studies, but also in the clinical trial space where such applications could be used for adverse event tracking and reporting.

A strength of our study is its prospective design, which avoids the problem of recall bias often associated with retrospective studies. The study follow-up period of one year also enabled the tracking of patients longitudinally over time, allowing for clearer evaluation of time to symptom resolution. Although the symptoms reported by patients were subjective in nature, the use of the validated SF12v2 health outcomes questionnaire allowed for a functional assessment of each study participant's overall mental health. Indeed, we showed that patients who reported persistence of fatigue and/or CNS-related symptoms beyond 3 months had a significantly lower MCS then their counterparts whose symptoms lasted less than 3 months, indicating that self-reported chronic sequelae translates into functionally worse mental health outcomes.

A limitation of our study is that patients in the ARI cohort were not tested specifically for DENV, and hence we are unable to completely exclude co-infection. We also acknowledge that the hospitalisation status was imbalanced between the two study cohorts, with a significantly higher proportion of the dengue cohort hospitalised at the time of enrolment, compared to the ARI cohort where the majority of patients were ambulatory. As such, we avoided making head-to head comparisons between the two cohorts.

In conclusion, we show that persistent fatigue and CNS-related chronic sequelae, as well as poorer overall mental health, occur in a significant proportion of both dengue and ARI patients post-infection. Our findings reveal an oft under-appreciated burden of post-infection

chronic sequelae in both dengue and ARI patients, and highlight the need for therapeutic and preventative strategies in order to prevent both acute viral infection and its associated chronic sequelae.

Acknowledgments

We thank the clinical research coordinators from the Department of Infectious Diseases, Singapore General Hospital and the Clinical Trials & Research Unit, Changi General Hospital for assisting with this study, and the patients who volunteered for the study. This work was funded by a SingHealth Foundation Research Grant (SHF/FG622P/2016) and the National Research Foundation through the Singapore MIT Alliance for Research and Technology Antimicrobial Resistance Integrative Research Group.

Figures and figure legends

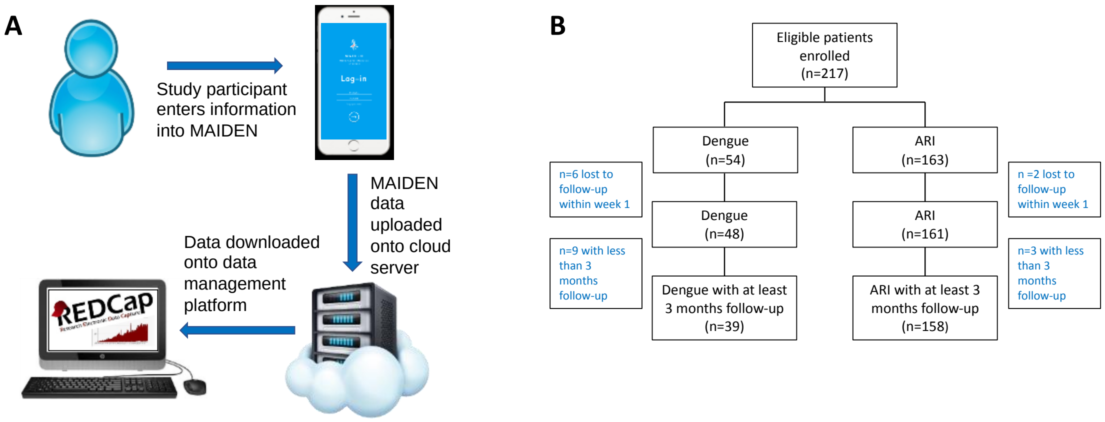

Fig 1. Study design and enrolment (A) Study data was collected using the Mobile Application for Information collection in Dengue (MAIDEN) application. At each study visit, patients would enter information remotely into MAIDEN which was installed on their mobile phone. The data collected would then be uploaded onto a secure cloud server and downloaded into the RedCap data management platform where it could be accessed by the study team. (B) Consort flow of patients enrolled onto the study. Patients were excluded from the overall analysis if they dropped out of the study or were lost-to-follow up within the first week after enrolment.

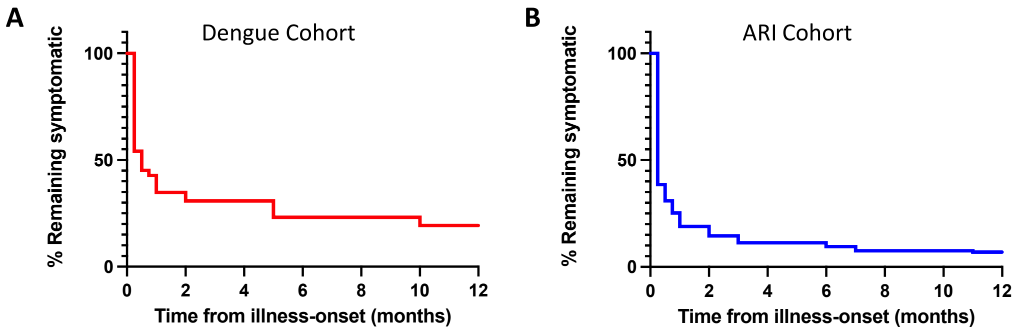

Months / No at risk	0	0.25	0.5	0.75	1	2	3	4	5	7	10	11	12
Dengue	48	48	24	19	16	9	9	8	8	8	6	6	4
ARI	161	161	61	49	40	30	23	23	18	15	15	12	11

Fig 2. Kaplan-Meier curve of time to symptom resolution. Each curve shows the overall time to symptom resolution (in months) after illness-onset in patients with (A) dengue and (B) acute viral respiratory infection (ARI). The numbers of patients at risk for each of these two groups are shown in the table immediately below the Kaplan-Meier curves.

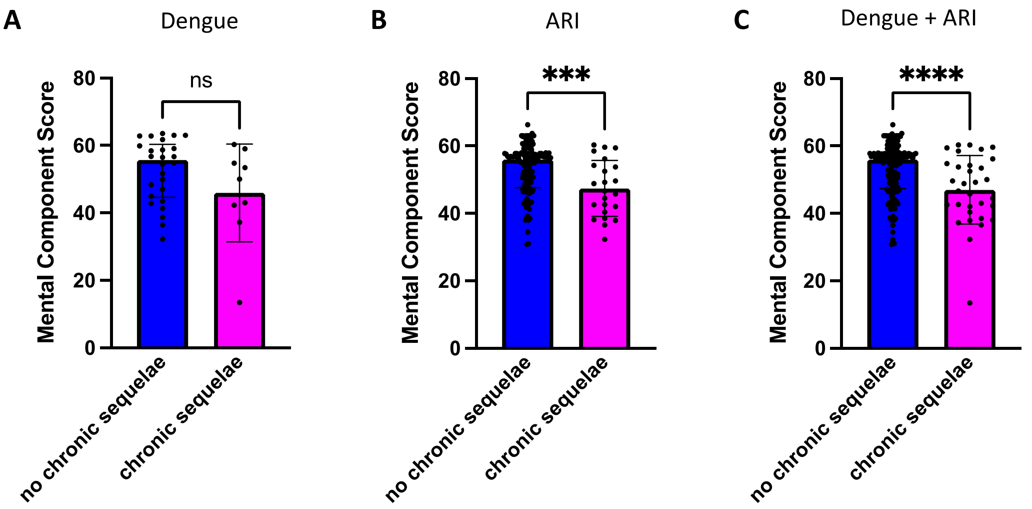

Fig 3. SF12v2 Mental Component Score (MCS) at month 3 in patients with and without chronic sequelae. (A) Comparison of MCS within the dengue cohort. (B) Comparison of MCS within the ARI cohort. (C) Comparison of MCS scores in both the dengue and ARI cohorts combined. Blue bars represent the group without chronic symptoms while magenta bars represent the group with chronic symptoms. Black dots indicate the individual MCS values. Summary plots show the median MCS with error bars representing the interquartile range. Statistics to compare the median MCS between groups was calculated using the Mann-Whitney test. *** = $p < 0.001$, ****= $p < 0.0001$, ns = not significant.

References

1. Michelen M, Manoharan L, Elkheir N, Cheng V, Dagens A, Hastie C, et al. Characterising long COVID: a living systematic review. BMJ Glob Health. 2021;6(9).

2. Nalbandian A, Sehgal K, Gupta A, Madhavan MV, McGroder C, Stevens JS, et al. Post-acute COVID-19 syndrome. Nat Med. 2021;27(4):601-15.

3. Su Y, Yuan D, Chen DG, Ng RH, Wang K, Choi J, et al. Multiple early factors anticipate post-acute COVID-19 sequelae. Cell. 2022;185(5):881-95.e20.

4. Hirschenberger M, Hunszinger V, Sparrer KMJ. Implications of Innate Immunity in Post-Acute Sequelae of Non-Persistent Viral Infections. Cells. 2021;10(8).

5. Guzman MG, Gubler DJ, Izquierdo A, Martinez E, Halstead SB. Dengue infection. Nature Reviews Disease Primers. 2016;2(1):16055.

6. Guo C, Zhou Z, Wen Z, Liu Y, Zeng C, Xiao D, et al. Global Epidemiology of Dengue Outbreaks in 1990–2015: A Systematic Review and Meta-Analysis. Frontiers in Cellular and Infection Microbiology. 2017;7.

7. Thomas SJ, Yoon IK. A review of Dengvaxia®: development to deployment. Hum Vaccin Immunother. 2019;15(10):2295-314.

8. Seet RC, Quek AM, Lim EC. Post-infectious fatigue syndrome in dengue infection. J Clin Virol. 2007;38(1):1-6.

9. García G, González N, Pérez AB, Sierra B, Aguirre E, Rizo D, et al. Long-term persistence of clinical symptoms in dengue-infected persons and its association with immunological disorders. Int J Infect Dis. 2011;15(1):e38-43.

10. Gonzalez D, Martinez R, Castro O, Serrano T, Portela D, Vazquez S, et al. Evaluation of Some Clinical, Humoral and Imagenological Parameters in Patients of Dengue Haemorrhagic Fever Six Months after Acute Illness. 2005.

11. Halsey ES, Williams M, Laguna-Torres VA, Vilcarromero S, Ocaña V, Kochel TJ, et al. Occurrence and correlates of symptom persistence following acute dengue fever in Peru. The American journal of tropical medicine and hygiene. 2014;90(3):449-56.

12. Teixeira LAS, Nogueira F, Nascentes GAN. Prospective study of patients with persistent symptoms of dengue in Brazil. Rev Inst Med Trop Sao Paulo. 2017;59:e65.

13. Tiga DC, Undurraga EA, Ramos-Castañeda J, Martínez-Vega RA, Tschampl CA, Shepard DS. Persistent Symptoms of Dengue: Estimates of the Incremental Disease and Economic Burden in Mexico. The American journal of tropical medicine and hygiene. 2016;94(5):1085-9.

14. Cheak-Zamora NC, Wyrwich KW, McBride TD. Reliability and validity of the SF-12v2 in the medical expenditure panel survey. Qual Life Res. 2009;18(6):727-35.

15. Chariyalertsak S, Wansom T, Kawichai S, Ruangyuttikarna C, Kemerer VF, Wu AW. Reliability and validity of Thai versions of the MOS-HIV and SF-12 quality of life questionnaires in people living with HIV/AIDS. Health Qual Life Outcomes. 2011;9:15.

16. Cernin PA, Cresci K, Jankowski TB, Lichtenberg PA. Reliability and validity testing of the short-form health survey in a sample of community-dwelling African American older adults. J Nurs Meas. 2010;18(1):49-59.

17. Bohannon RW, Maljanian R, Landes M. Test-retest reliability of short form (SF)-12 component scores of patients with stroke. Int J Rehabil Res. 2004;27(2):149-50.

18. WHO Guidelines Approved by the Guidelines Review Committee. Dengue: Guidelines for Diagnosis, Treatment, Prevention and Control: New Edition. Geneva: World Health Organization

Copyright © 2009, World Health Organization.; 2009.

19. Mandarano AH, Maya J, Giloteaux L, Peterson DL, Maynard M, Gottschalk CG, et al. Myalgic encephalomyelitis/chronic fatigue syndrome patients exhibit altered T cell metabolism and cytokine associations. J Clin Invest. 2020;130(3):1491-505.

20. Brenu EW, Hardcastle SL, Atkinson GM, van Driel ML, Kreijkamp-Kaspers S, Ashton KJ, et al. Natural killer cells in patients with severe chronic fatigue syndrome. Auto Immun Highlights. 2013;4(3):69-80.

21. Klimas NG, Salvato FR, Morgan R, Fletcher MA. Immunologic abnormalities in chronic fatigue syndrome. Journal of clinical microbiology. 1990;28(6):1403-10.

22. Crook H, Raza S, Nowell J, Young M, Edison P. Long covid-mechanisms, risk factors, and management. BMJ (Clinical research ed). 2021;374:n1648.

23. Number of smartphone subscritpions wordlwide from 2016 to 2027 [Available from: https://www.statista.com/statistics/330695/number-of-smartphone-users-worldwide/.

Click here to access/download
Supporting Information
Supporting Information.docx
