# Supplementary material for: Chronic sequelae complicate convalescence from both dengue and acute viral respiratory illness
Source: PLoS Negl Trop Dis. 2022 Aug 18;16(8):e0010724. doi: 10.1371/journal.pntd.0010724 (PMC9426910; doi:10.1371/journal.pntd.0010724)
Supplement: Attachment — Submitted filename: Response to reviewers.docx [file pntd.0010724.s003.docx]

**Response to Reviewers**

We thank the reviewers for their insightful comments and helpful suggestions to improve the quality of our manuscript. We have taken these comments into consideration and have revised our manuscript accordingly.

We provide a point-by-point response to the reviewers’ individual comments below.

Reviewer 1

1. The manuscript discussed the post-infection long sequelae, on both dengue and acute respiratory infection (ARI), to evaluate the post-infection symptoms and its impact on patients' mental health. The study is clearly defined, with appropriate objective and hypothesis. The study design is suitable to answer the research question. The population is sufficient, with limitation has already been mentioned and acknowledged by the authors. The sample size might be underwhelming, as the prevalence of both diseases are massive in Singapore, however, I would believe it is still acceptable. The statistical analysis was also appropriate. Overall, I would deem the study is well-written, with appropriate design.

We thank the reviewer for their overall positive comments about our work.

2. I can't find the use of SF12v2 result on the result section; however, it seems to be used on Figure 3, I would suggest adding more elaborate explanation on this. Thus, the readers would be clear on the comparison of both populations. More elaborate discussion on this topic would also be more appreciated.

We thank the reviewer for pointing out this omission and have amended the paragraph in the results section referencing Figure 3 to read as follows:

“In order to determine if self-reported symptoms of chronic sequelae translated into worse mental health outcomes, we compared the SF12v12 MCS scores at month 3 in patients with and without chronic sequelae. In the dengue and ARI cohorts combined, the median month 3 MCS was lower in patients who remained symptomatic at 3 months and beyond, compared to those whose symptoms fully resolved (47.7 vs. 56.0, p<0.001) (Fig 3A), indicating that patients who self-reported persistence of symptoms beyond 3 months also experienced functionally worse mental health. This difference in the median month 3 MCS scores between those with and without chronic sequelae was also observed in the ARI cohort alone (46.6 vs. 56.0, p<0.01) (Fig 3B). Although a similar trend was observed in the dengue cohort, this did not reach statistical significance, possibly due to the smaller sample size in this cohort (50.0 vs. 55.7, p=0.20) (Fig 3C).”

We have also amended line 35 of the Abstract to read as follows:

“The median month-3 SF-12v2 Mental Component Summary Score was lower in patients who remained symptomatic at 3 months and beyond, compared to those whose symptoms fully resolved (47.7 vs. 56.0, p<0.001).”

3. The conclusions are well presented, and limitation has already been acknowledged by the authors. The data can be used to properly evaluate the post infection sequelae that might be found in both dengue and ARI patients, to support whole comprehensive treatment. The relevance to public health is highly correlated.

We thank the reviewer for highlighting the relevance of our work to public health.

4. Data of MSC or SF12v2 should be added to the manuscript. Otherwise, the data is well presented.

We thank the reviewer for highlighting this omission and have addressed their suggestion in our reply to Point 1.

Reviewer 2

1. This study has been well planned, with validated and well characterized study tools used and patients have been prospectively followed up. Symptoms have been recorded using a mobile app, so that symptoms are not missed. However, the main limitation of this study is the small sample size of patients with dengue (n=48), with only one patient having severe dengue. While the results highlight some important findings, it would be very interesting to see the results in a larger sample size. This study has been compared with the chronic sequel of COVID-19. In such studies, the sample sizes have been extremely large. Therefore, it would be important to see these results in a larger study population as well, in patients with varying clinical disease severity. It would be best if the authors discuss the limitations in their sample size in the discussion. There are no concerns regarding ethical or regulatory requirements.

The study is well planned and executed, with appropriate data analysis plan. However, the sample size is quite small.

We thank the reviewer for their overall positive comments We acknowledge the relatively small sample size as a limitation of our study and have amended the discussion section to include this:

“…we acknowledge that our study sample size was relatively small, particularly in the dengue cohort, as case numbers in Singapore during the study enrolment period were low. Nevertheless, we believe that the robustness of our study design and data collection methods have allowed for a rigorous assessment of post-infective chronic sequelae in our study cohort.”

2. The authors state that ‘Apart from this report, no other studies to date explored the causes of post dengue chronic sequelae’ : However, this statement is not very accurate as there have been studies looking at chronic sequel, although not using the same study tools as described in this study. It would be important to discuss the following studies and how these previous studies relate to this study.
   • 2 month follow up study of 177 patients (Seet et al, J Clin Virol 2007)
   •  Cost of persistent dengue: Persistent Symptoms of Dengue: Estimates of the Incremental Disease and Economic Burden in Mexico (Tiga et al, Am J Trop Med Hyg 2016)
   •  2 month follow up study of 158 patients (Sigera et al, Trans R Soc Trop Med Hyg 2021)

We thank the reviewer for highlighting these three studies, and indeed we have already cited the studies by Seet et al and Tiga et al in our introduction section discussing existing studies in the literature, and their limitations (see paragraph 2 of Introduction section). We have now also included the study by Sigera et al (new Ref No: 13).

We should clarify that our statement “*Apart from this report, no other studies to date explored the causes of post-dengue chronic sequelae*” was not referring to our study but rather to the study by Garcia G et al, Int J Infect Dis 2011 which has been one of the only studies to date to look specifically at possible mechanistic causes of post-dengue chronic sequelae. The other 3 studies highlighted by the reviewer explored the prevalence of post-dengue chronic sequelae and its associations but do not study causal factors.

3. The authors discuss the possible causes of chronic sequel of dengue. It would be also important to discuss long term autoimmune neurological diseases and other autoimmune diseases following dengue.

Pattern Recognition Approach to Brain MRI Findings in Patients with Dengue Fever with Neurological Complications (Vyas et al, Neurol India 2020).

We thank the reviewer for the suggestion and have now cited the findings from this paper in paragraph 5 of the discussion section:

“… structural brain changes on magnetic resonance imaging have been found in patients with post-dengue neurological sequelae such as encephalopathy, suggesting a pathological basis for such sequelae (Vyas et al, 2020)”

In the discussion session, we have highlighted autoimmunity as a potential cause of chronic sequelae, both in dengue as well as in other viral infections. However, we feel that a detailed discussion of autoimmune disease post-dengue is beyond the scope of this paper.

Reviewer 3

1. How could you distinguished between symptom occurred at 3 months or during monitoring were belongs to dengue not belongs to other acute symptoms or conditions?
   Authors also included patients with co-morbidities, those co-morbidities can contribute to symptoms during monitoring. Do you have records of any acute symptoms (for example, another dengue infection, another ARI) during monitoring i.e fever, diarrhoea, cough, sneezing, myalgia. If you had, did you do laboratory testing to find out the possible cause of aetiology?

Authors should described more about limitation that study could not absolutely sure that the symptoms occurred during monitoring may belongs to other disease/condition.

We thank the reviewer for the comment. All the symptoms we analysed were those that the patients complained of during the acute phase of illness and which then continued through fever defervescence and convalescence. Symptoms that resolved and then subsequently appeared again after more than a 2-month period (as described in the Definition subsection of the Methods section) were excluded. This would have made it less likely that such symptoms were due to other acute illnesses that developed during the period of follow-up.

To clarify further, we have added the following text to paragraph 5 of the Discussion section:

“In our analysis, we did not include symptoms that resolved and then re-occurred after more than a two-month period in order to minimize the possibility that such symptoms were due to other acute illnesses.”

With respect to co-morbidities, the percentage of patients with co-morbidities such as diabetes and IHD were less than 5% in both groups, and less than 10% for hypertension. These rates are comparable in both the dengue and ARI groups. If indeed the chronic symptoms were driven by co-morbidities, then we should not have observed a higher rate of chronic sequelae in dengue compared to the ARI subjects. Furthermore, we were also careful to exclude any patients with other underlying chronic illnesses such as neurological, rheumatological or psychiatric disease, dementia or cognitive impairment, malignancy or recent surgery (as described in the Methods section). Hence it is unlikely that co-morbidities would account for the prevalence of chronic sequelae observed.

2. The analysis presented match the analysis plan. The results are clearly presented.

We thank the reviewer for their positive comments.

Other amendments

1. We have included an “Author Summary” after the Abstract as per journal requirements.
2. We have corrected the term “Mental Component Score” to “Mental Component Summary Score”.
3. We have modified Figure 1A to use open source Clip Art instead, so as to not infringe copyright laws.
4. We have changed the word “group” to “cohort” in Tables 2 and 3 so as to standardise with the rest of the manuscript.
5. We have amended Figure 3 to show the combined dengue and ARI cohorts results first i.e. following the sequence that the results are described in the main text.
6. We have made corrections to the Supporting Information S1 Study Questionnaire to include the SF12v2 questions (these were unintentionally omitted in or original submission).
